# Supplementary material for: A genetically attenuated malaria vaccine candidate based on P. falciparum b9/slarp gene-deficient sporozoites
Source: eLife. 2014 Nov 19;3:e03582. doi: 10.7554/eLife.03582 (PMC4273440; doi:10.7554/eLife.03582)
Supplement: Supplementary file 1. — Oocyst and sporozoite production and sporozoite characteristics (motility, traversal, hepatocyte invasion) of the P. berghei mutants PbΔslarp and PbΔb9Δslarp. DOI: http://dx.doi.org/10.7554/eLife.03582.011 [file elife03582s001.docx]

**Supplementary File 1:** Oocyst and sporozoite production and sporozoite characteristics (motility, traversal, hepatocyte invasion) of the *P. berghei* mutants Pb∆*slarp* and Pb∆*b9*∆*slarp*

| **Parasite** | **Oocyst no.^a^** | **Sporozoite no.^b^** | **Gliding motility^c^** | **Cell traversal^d^** | **Hepatocyte invasion^e^** |  |
| --- | --- | --- | --- | --- | --- | --- |
|  | **Mean ± sd** | **Mean ± sd** | **Mean ± sd** | **Mean ± sd** | **Mean ± sd** |  |
|  |  |  |  |  |  |  |
| PbWT (GFP-Luc_con_) | 204 ± 113 | 98K ± 26K | 70.3 ± 9 | 19.9 ± 2.5 | 42.3 ± 8.2 |  |
| PbΔ*slarp* | 221 ± 61 | 88K ± 16K | 76 ± 10 | 21.7 ± 1.3 | 39.3 ± 3.1 |  |
| PbΔ*b9Δslarp* | 176 ± 131 | 104K ± 30K | 69 ± 4 | 24.1 ± 2.6 | 43.5 ± 4.2 |  |
| ^a^ Mean number of oocysts per mosquito | | |  |  | |  |
| ^b^ Mean number of sporozoites per salivary gland | | |  |  | |  |
| ^c^ percentage of sporozoites that show gliding motility. | | | |  | |  |
| ^d^ Percentage of dextran positive hepatocytes | | |  |  | |  |
| ^e^ Percentage of intracellular sporozoites at 3 hours post infection of hepatocytes | | | | | |  |
